# Management of a COVID-19 outbreak using a multidisciplinary approach and infection prevention control practices at a community living center in Veterans Administration Hospital, North Texas

**DOI:** 10.1017/ash.2024.491

**Published:** 2025-01-17

**Authors:** Ikwo K. Oboho, John Hanna, Denisse Silva-Rodriguez, Angela Christie-Smith, Andrew Psenicka, Ampava Khongmongkhon, Marcus A. Kouma, Sherry Reid, Roger Bedimo

**Affiliations:** 1 Division of Infectious Diseases & Geographic Medicine, University of Texas Southwestern Medical Center, Dallas, TX, USA; 2 Veterans Affairs North Texas Health Care System, Dallas, TX, USA; 3 East Carolina University (ECU) Health, Greenville, NC, USA; 4 Brody School of Medicine, ECU, Greenville, NC, USA; 5 Walden University, Dallas, TX, USA

## Abstract

**Background:**

The increase in severe acute respiratory coronavirus virus 2 (SARS-CoV-2) cases due to the omicron strain led to reduced acute care hospital beds at the Veterans Administration (VA) Hospital, North Texas; veterans with non-severe coronavirus disease 2019 (COVID-19) disease were managed at a community living center (CLC), a VA nursing home. The management of non-severe COVID-19 in VA nursing homes has not been extensively described.

**Methods:**

We describe resident characteristics and outcomes, and infection control practices implemented during 2 COVID-19 outbreak periods (January 12–February 15, 2022, June 28–July 14, 2023). Serial testing of all CLC residents was conducted, and residents with polymerase chain reaction-confirmed SARS-CoV-2 (COVID-19) infection were included in the analysis. Resident data were ascertained from the COVID-19 facility dashboard and medical record system.

**Results:**

From January 12 to February 15, 2022, and June 28–July 14, 2023, 62 adults residing at the CLC were diagnosed with COVID-19. Overall, the median age was 75 years [interquartile range, 71–80], and 57 (91.9%) were men. Residents were cohorted by COVID-19 test results. A multidisciplinary team was convened, and staff were fit tested for appropriate personal protective equipment (PPE) and received refresher training on hand hygiene, donning, and doffing of PPE. Thirty-seven (59.7%) residents were symptomatic. Overall, 55 (88.7%) residents were documented to have received the SARS-CoV-2 primary vaccination series. Most residents were managed at the CLC, while 12 (19.3%) were hospitalized in acute care.

**Conclusions:**

It is feasible to manage high-risk residents with non-severe COVID-19 disease in a CLC utilizing a multidisciplinary approach and implementing Infection Prevention and Control strategies.

## Background

During the coronavirus disease 2019 (COVID-19) pandemic, persons aged 65 and older and those with one or more underlying medical conditions experienced a disproportionately high hospitalization rates and mortality.^1–3^ In addition, persons in long-term care facilities also experienced a high number of deaths given the prevalence of elderly patients with multiple comorbid conditions. According to one study, an estimated 21% of COVID-19-related deaths occurred among nursing home residents.^4^ Crisis standards of care have been established, which recommend considerations for alternate care systems such as during pandemics.^5^

During the early period of the pandemic (2020–2021), residents who tested positive for SARS-CoV-2 were transferred to the acute care hospital. In 2022, the SARS-CoV-2 omicron variant strain resulted in a large increase in cases and significant utilization of healthcare resources, which reduced the acute care hospital capacity at the Veterans Administration Hospital, North Texas Health Care System (VANTHCS). As a result, veterans with non-severe diseases were managed at a VANTHCS community living center (CLC), a Veterans Administration (VA) nursing home during 2 separate COVID-19 outbreaks. We describe resident characteristics and outcomes, infection control practices implemented, and lessons learned from managing patients at the CLC during 2 separate SARS-CoV-2 outbreaks in 2022 and 2023.

## Methods

The CLC is a VA nursing home where residents can stay for a short time or for an extended duration. At the CLC, residents can receive a nursing home level of care including assistance for activities of daily living (such as bathing and getting dressed) and skilled nursing and medical care including wound care or intravenous antibiotics. Additional services offered at the CLC include sub-acute rehabilitation, mental health recovery care, special care for veterans with dementia or other cognitive deficits, respite care, palliative care, and hospice care for end-of-life care. The Bonham VA CLC, where the outbreak occurred, is located nearly 4 hours away from the main Dallas VA Medical Center, and the usual patient census ranges from 90 to 100 residents. Residents at the CLC with polymerase chain reaction (PCR)-confirmed SARS-CoV-2 infection during the 2 large outbreaks from January 12 to February 15, 2022, and June 28–July 14, 2023, were included in the descriptive analysis. Resident data were collected from the facility’s COVID-19 dashboard and medical record system.

VANTHCS developed protocols for testing and isolation in March 2020 to decrease the risk of transmission to Veteran populations. Prior to January 2022, residents who tested positive for COVID-19 or had high-risk exposures were transferred to a designated COVID-19 unit, within the acute care unit of the Dallas VA Medical Center. From January 2022 and during large outbreaks, COVID-19 patients with mild to moderate disease were managed at the CLC. In addition, persons under investigation (PUI) who were exposed to residents diagnosed with COVID-19, were also isolated and observed with serial testing at the CLC. Prior to the SARS-CoV-2 Omicron wave in 2022, residents at the CLC and staff providing care or services in the CLC were tested twice weekly using PCR testing. During the outbreak period, testing frequency was increased to 3 times weekly.

A team of geriatrics and extended care (GEC) providers including the CLC pharmacist and nurse practitioner reviewed the clinical profile of residents with positive COVID-19 tests and classified them by severity criteria and the presence of underlying comorbidities and high-risk conditions. This classification was verified by the CLC Physicians. COVID-19 severity criteria were adapted from the National Institutes of Health (NIH) COVID-19 treatment guidelines and classified as mild, moderate, or severe.^6^ Mild illness was defined as any of the various signs and symptoms of COVID-19 (eg, fever, cough, sore throat, malaise, headache, muscle pain) without shortness of breath, dyspnea, or abnormal chest imaging. Moderate illness was defined as any evidence of lower respiratory disease by clinical assessment or imaging, and a saturation of oxygen (SpO2) ≥92%. Veterans who were determined to have mild to moderate disease were deemed stable for treatment at the CLC. Veterans with oxygen saturation <92% were considered to have severe disease which required immediate notification of GEC providers for review and additional measures were implemented for escalation of care to the Dallas VA acute care hospital. The threshold of 92% was chosen because of the high prevalence of underlying chronic lung disease in this population. GEC medical providers and pharmacists identified eligible patients for COVID-19 therapies after discussion with infectious diseases specialists and pharmacists based on the VANTHCS COVID-19 treatment guidelines. No treatment was recommended for asymptomatic patients per the treatment guidelines, but asymptomatic veterans with high-risk conditions could also be prescribed COVID-19 antivirals based on GEC provider decisions in consultation with ID specialists. Remdesivir was the only antiviral available for treatment during the first outbreak. For the second outbreak, symptomatic residents could receive Paxlovid, remdesivir, or molnupiravir as antivirals based on the symptom duration and presence of high-risk conditions according to NIH treatment guidelines.^6^ Residents were reviewed for eligibility for Paxlovid; if ineligible, this was followed by remdesivir and then molnupiravir. Nursing staff were trained on COVID-19 treatment protocols. For the outcome variables, hospitalization status required at least an overnight admission at the acute care hospital. Death was defined as death within 30 days of a COVID-19-positive test for veterans admitted to acute care hospital or who remained at the CLC. Intensive care unit (ICU) status required patients to receive ICU-level care. The project was reviewed and deemed as quality improvement and designated as nonresearch; therefore, Institutional Review Board approval was waived.

## Results

### Infection prevention practices

For the initial outbreak in 2022, a multidisciplinary team was convened consisting of representatives from Nursing Service, Infection Prevention and Control (IPC), Safety, Engineering, Nutrition and Food Service, GEC providers, Emergency Operations Center, Environmental Management Services, Physical Medicine and Rehabilitation, Supply Chain Management, Recreation Therapy, Pathology and Laboratory Medicine, and Psychology to formulate plans and ensure resources were available for veterans. In addition, CLC staff were fit tested for appropriate personal protective equipment (PPE) and received refresher training on hand hygiene, donning and doffing of PPE. Residents were cohorted based on SARS-CoV-2 results. Veterans with PCR-confirmed tests for SARS-CoV-2 were moved to designated areas within the CLC for positive veterans, and those with initial negative tests and/or high-risk exposures were placed in an observation area until a confirmatory PCR test was performed. Designated areas were converted to COVID-19 treatment units, and a separate area was designated for PUIs with high-risk exposures and negative SARS-CoV-2-negative test results.

Given the shortage of beds in acute care, a multidisciplinary team was convened to create a designated COVID unit in the CLC for cohort isolation of COVID-19-positive residents in a designated unit/hallway. The choice of the location of the COVID units considered unit ventilation capabilities including discussions with engineering about airflow/ventilation and heating, ventilation and air conditioning (HVAC) systems, unit isolation abilities, location of medication rooms, and ability to limit staff flow between COVID and non-COVID units. GEC leadership, Engineering, Patient Safety, and IPC reviewed the Veterans Health Administration (VHA) HVAC Design Manual Guidance for the CLC. Engineering recommended not increasing the air exchanges for the entire Bonham CLC because of concerns that it could alter the building airflow and potentially lead to mechanical failures given that the Bonham CLC is an older building. The air exchanges at the CLC ranged from 4 to 10 air changes per hour (ACH) and met CLC standards referenced in the HVAC Design Manual. There were no additional changes made to airflow in the CLC. Even though this did not meet the minimum recommended ACH for negative pressure rooms, additional mitigation measures were taken including minimizing aerosol exchange activities, switching veterans on nebulizers to metered dose inhalers, and ensuring the door was kept shut for rooms with COVID-19-positive veterans.

Interdisciplinary calls between services were conducted twice daily to review PPE supplies, barriers to practice, staffing capacity and address implementation challenges. Physical building evaluations to address ventilation were implemented, and environmental sanitation staffing was reinforced. In addition, breakroom locations were created to minimize interaction between staff working on the COVID and non-COVID units. Facility staff wore appropriate N95 respirators and adhered to extended droplet and contact precautions (airborne plus contact precautions) for suspected/known COVID-19-positive residents. Nursing managers were integral in ensuring that residents were cohorted accordingly and residents and staff were screened and tested in a timely manner. Collaboration between services and daily team huddles was an integral part of daily operations. In addition, during the outbreak period, dining room service and other communal activities were halted; admissions were also halted, except for admissions for end-of-life care. Similar procedures were adopted for the second outbreak at the Bonham CLC in 2023.

### Patient characteristics and outcomes

Overall, demographic characteristics (age, sex, and race) were similar during both outbreaks (Table 1). Most residents were male (91.9%) and white (77.4%). Among the 62 residents who tested positive during both outbreak periods, 37 (59.7%) were symptomatic, and over a third of veterans had at least 3 comorbidities. Nearly 90% of residents had received their primary COVID-19 vaccination series. Twelve (19.3%) veterans were hospitalized outside the CLC at the acute care hospital, and 1 (1.6%) resident died.

**Table 1.** Epidemiological characteristics and outcomes of laboratory-confirmed COVID-19 cases at the community living center, Veterans Administration Hospital, North Texas Health Care System, outbreak 1, January–February 2022, (N = 33), outbreak 2, June–July 2023 (N = 29)

Characteristics	Total N=62	Outbreak 1, N=33: n (%)	Outbreak 2, N=29: n (%)
**Location**			
Unit A	19 (30.6%)	19 (57.6%)	0 (0.0%)
Unit B	34 (54.8%)	5 (15.2%)	29 (100.0%)
Dementia unit	9 (14.5%)	9 (27.3%)	0 (0.0%)
**Age in years, median [IQR]**	75 [69–80]	76 [71–80]	75 [67–80]
**Male sex**	57 (91.9%)	30 (90.9%)	27 (93.1%)
**Race**			
White	48 (77.4%)	25 (75.8%)	23 (79.3%)
Black/African American	10 (16.1%)	5 (15.2%)	5 (17.2%)
Unknown	3 (4.8%)	2 (6.1%)	1 (3.4%)
Asian	3 (4.8%)	1 (3.0%)	0 (0%)
**BMI (kg/m^2^)**			
18.5–24.9 (healthy weight)	16 (25.8%)	7 (21.2%)	9 (31.0%)
25–29.9 (overweight)	22 (35.5%)	15 (45.5%)	7 (24.1%)
≥30 (obesity)	24 (38.7%)	11 (33.3%)	13 (44.9%)
**Comorbidity**			
Ischemic heart disease	20 (32.3%)	8 (24.2%)	12 (41.4%)
Stroke	15 (24.2%)	6 (18.2%)	9 (31.0%)
COPD	12 (19.4%)	6 (18.2%)	6 (20.7%)
ESRD	4 (6.5%)	2 (6.1%)	2 (6.9%)
PVD	7 (11.3%)	2 (6.1%)	5 (17.2%)
Other^1^	28 (45.2%)	2 (6.1%)	26 (89.7%)
**Number of comorbidities**			
0	17 (27.4%)	17 (51.5%)	0 (0.0%)
1	15 (24.2%)	9 (27.3%)	6 (20.7%)
2	7 (11.3%)	5 (15.2%)	2 (6.9%)
3+	23 (37.1%)	2 (6.1%)	21 (72.4%)
**COVID-19 vaccination**			
Primary series	55 (88.7%)	28 (84.8%)	27 (93.1%)
Vaccinated and boosted	46 (74.2%)	24 (72.7%)	22 (75.9%)
**Treatment**^2^			
**Symptomatic status**	**37 (59.7%)**	**23 (69.7%)**	**14 (48.3%)**
Remdesivir	19 (51.3%)	17 (73.9%)	2 (14.3%)
Paxlovid	10 (27.0%)	0 (0.0%)	10 (71.4%)
Dexamethasone	11 (29.7%)	11 (47.8%)	0 (0.0%)
**Asymptomatic status**	**25 (40.3%)**	**10 (30.3%)**	**15 (51.7%)**
Remdesivir	9 (36.0%)	8 (80.0%)	1 (6.7%)
Paxlovid	6 (24.0%)	0 (0.0%)	6 (40.0%)
Dexamethasone	0 (0.0%)	0 (0.0%)	0 (0.0%)
**Outcomes**			
Hospitalized outside CLC	12 (19.3%)	7 (21.2%)	5 (17.2%)
ICU	0 (0%)	0 (0%)	0 (0%)
Death	1 (1.6%)	1 (3.0%)	0 (0%)

Note. BMI, body mass index; COPD, chronic obstructive pulmonary disease; ESRD, end-stage renal disease; PVD, peripheral vascular disease.

1 Other comorbidity – outbreak 1: asthma n = 1 and chronic liver disease n = 2; outbreak 2: asthma n = 2, chronic liver disease n = 2, diabetes n = 14, hypertension n = 26, % represent a subset of the total denominator from both outbreaks (N) or outbreaks 1 or 2.

2 Treatment % for symptomatic and asymptomatic status calculated out of a denominator of overall outbreak total and individual outbreaks. Subgroup treatment % by symptomatic and asymptomatic status was calculated out of the denominator of symptomatic and asymptomatic residents by the outbreak.

**Figure 1.**
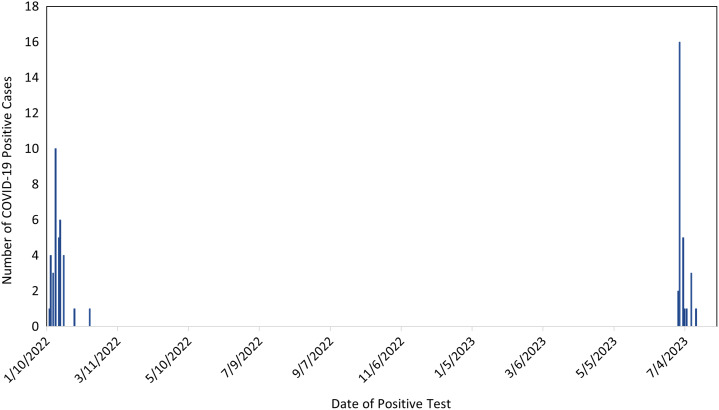
Epidemic curve of laboratory-confirmed coronavirus disease 2019 (COVID-19) disease at a community living center, Veterans Administration Hospital, North Texas Health Care System, January–February 2022, June–July 2023.

For the first outbreak at Bonham CLC, which occurred from January 12 to February 15, 2022, 33 (37.5%) of 88 residents at the Bonham CLC tested positive for SARS-CoV-2. The outbreak affected 3 different units in the CLC, and most infections (93.9%) occurred between January 12 and 24 (Fig. 1). The median age was 76 years [interquartile range, 71–80 years]; 30 (90.9%) were men, 25 (75.8%) were white, and 5 (15.2%) African American (Table 1). Twenty-three of 33 (69.7%) residents were symptomatic. Overall, 28 (84.8%) were documented to have received their primary COVID-19 vaccination series, and 24 (72.7%) had received recommended COVID-19 boosters at the time of the outbreak. Ischemic heart disease, chronic obstructive pulmonary disease, and stroke were the most common comorbidities. For management of COVID-19, out of 21 symptomatic veterans, 17 (80.9%) received remdesivir and 11 (52.3%) received dexamethasone. Reasons for refusal of treatment with remdesivir for outbreak 1 included apprehension about taking a new medication, veteran’s hospice status, and requirement for intravenous access for remdesivir for a veteran with dementia and combative behavior. Out of 10 asymptomatic residents, 8 (80%) residents received remdesivir because of underlying high-risk conditions. Most residents were determined to have mild or moderate COVID-19 and managed at the CLC, while 7 (21.2%) were hospitalized in the acute care hospital. No residents required ICU admission. Overall, 32 (97%) residents survived, while 1 resident died. The resident who died remained at the CLC and had an underlying diagnosis of end-stage dementia and failure to thrive. The resident died within 2 weeks of the COVID-19 diagnosis from COVID-19-related pneumonia and was transitioned to hospice care 48 hours before death.

For the second outbreak, which occurred from June 28–July 14, 2023, 29 (29.3%) of 99 residents at the Bonham CLC tested positive for SARS-CoV-2 (Fig. 1); the outbreak affected only 1 unit and lasted a shorter duration than the 2022 outbreak. Demographic characteristics (age, sex, and race) were similar to the first outbreak (Table 1). Fourteen of 29 (48.2%) residents were symptomatic. Similar to the first outbreak, ischemic heart disease, chronic obstructive pulmonary disease, and stroke were the most common comorbidities. For the management of COVID-19, out of the 14 symptomatic veterans, 10 (71.4%) received Paxlovid, 2 (14.3%) received remdesivir, and no one received dexamethasone. Remdesivir or Paxlovid was not prescribed for 2 symptomatic veterans with underlying high-risk medical conditions because they had mild symptoms (cough, nasal congestion). Out of 15 asymptomatic residents, 7 (46.7%) received COVID-19 antivirals: 6 received Paxlovid and 1 received remdesivir. Most residents were managed at the CLC, while 5 (17.2%) were hospitalized in the acute care hospital. No veterans required ICU admission nor died during outbreak 2.

Comparing both outbreaks, more residents were symptomatic during the first outbreak compared to the second outbreak (23 of 33 [69.7%] vs 14 of 29 [48.3%]). Coverage of the primary COVID-19 vaccine series was lower during the first outbreak compared to the first outbreak (84.8% vs 93.1%). Fewer residents had at least 3 comorbidities during the first compared to the second outbreak (6.1% vs 72.4%). In addition, a smaller proportion of the residents were obese in the first outbreak compared to the second outbreak (33.3% vs 44.9%). More symptomatic residents received remdesivir during the first compared to the second outbreak (17 of 23 [73.9%] vs 2 of 14 [14.3%]) while nirmatrelvir-ritonavir (Paxlovid) was the prevalent antiviral used during the second outbreak (10 of 14, 71.4%) among symptomatic residents. In addition, more asymptomatic residents received antivirals during outbreak 1 (8 of 10 [80.0%] vs. 7 of 15 [46.7%]) compared to outbreak 2. No residents received molnupiravir as veterans were able to receive remdesivir and/or Paxlovid, in accordance with the FDA-issued Emergency Use Authorization (EUA) for molnupiravir for use when other FDA-approved or authorized treatment options are not available or are contraindicated. A larger proportion of residents were hospitalized outside the CLC during the first compared to the second outbreak (21.2% vs 17.2%). No residents required ICU admission in both outbreaks, and only 1 resident died in outbreak 1 compared to no deaths in outbreak 2.

## Discussion

Our findings demonstrate that it is feasible to deliver care to COVID-19 residents in the CLC including COVID-19 antivirals to high-risk residents with stable disease with a multidisciplinary team and IPC strategies. Our population had a high COVID-19 vaccination coverage and nearly 75% of residents were managed at the CLC: there was only 1 death. In addition to the COVID-19 vaccination of residents, we implemented testing strategies for early identification of positive residents and used nonpharmacologic outbreak control measures to supplement pharmacologic measures to mitigate the outbreak in this high-risk population. Remdesivir was administered to nearly 75% of symptomatic residents during the first outbreak, while both Paxlovid and remdesivir were administered to approximately 85% of symptomatic residents during the second outbreak.

COVID-19 had a significant impact on long-term care facilities and caused significant morbidity and mortality.^7^ The VHA issued guidance to mitigate this morbidity.^8^ The use of the VA CLC for housing clinically stable COVID-19 patients has previously been described during the early period of the COVID-19 pandemic in 2020.^9^ In that publication, a COVID-19 post-acute care unit was created within a traditional long-term care unit near the Department of VA Greater Los Angeles Healthcare system for patients who were transferred from the acute care hospital to a COVID recovery unit when they were deemed clinically stable, as a step-down model of care. In contrast to that setting, we describe a setting where residents were not previously admitted to the acute care hospital and one in which the CLC was not contiguous to the acute care hospital which reinforced the need for a designated COVID-19 unit at the CLC. Building on that study, our findings add to the literature by describing patient comorbidities and the use of COVID-19 therapeutics in the CLC setting. In addition, we describe a secondary outbreak where lessons learned were further adapted and eligible residents received the oral COVID-19 antiviral, Paxlovid.

Furthermore, COVID-19 vaccination coverage was high among the CLC residents which may have contributed to the favorable outcomes we noted; this high vaccination coverage is not surprising since residents of long-term care facilities were recommended by the Advisory Committee on Immunization Practices to be offered COVID-19 vaccinations first in phase 1a of the vaccination program.^10^ Previous studies comparing the impact of COVID-19 vaccination have noted a reduction in mortality over time across all age groups including the elderly.^11^ We may also have observed favorable outcomes because the outbreaks occurred in 2022 and 2023, several years after the initial onset of the pandemic in a highly vaccinated population who likely also had hybrid immunity from prior COVID-19 infections. In addition, the use of antivirals including remdesivir and Paxlovid has been previously demonstrated to shorten the time to recovery and reduce the risk of hospitalization and death from COVID-19.^12–17^ In a VA study, among outpatients, compared with matched untreated controls, those treated with nirmatrelvir-ritonavir paxlovid during the Omicron variant transmission period had a lower-30-day risk for hospitalization and death.^17^

Our findings have several limitations. First, we did not assess resident experience during the outbreaks and it’s unclear whether eliminating the long transportation to the acute care hospital improved resident experiences. Second, because we did not perform additional laboratory testing for genetic sequencing, we are unable to ascertain the relatedness of the COVID-19 strains responsible for the outbreaks among veterans and provide a detailed description of person-to-person transmission during the outbreak. Third, we did not assess the potential contribution of transmission of COVID-19 infection from staff to veterans during the outbreak. The implementation of IPC, medical and environmental interventions, and early identification and cohort isolation was instrumental in mitigating this outbreak. Given the waning active and passive immunity from vaccination and prior COVID-19 infections, and the risk of repeated COVID-19 infections in this high-risk population, this model of care can be further adapted for future COVID-19 outbreaks at the CLC. The creation of a COVID-19 unit within the CLC decreased the transfer of CLC patients to the acute care hospital and reduced the pressure for acute care hospital beds.
